# Deep learning model based on DCE-MRI: fusion of 3D features of tumor, peritumoral vessels and metastatic lymph nodes for prediction of pathological complete response to neoadjuvant therapy in breast cancer

**DOI:** 10.3389/fonc.2025.1664631

**Published:** 2025-12-02

**Authors:** Yanchen Du, Yue Zhu, Mengting Yang, Aoqi Zhang, Lei Zhang, Zhihuai Zhou

**Affiliations:** 1 Department of Radiology, The Second Affiliated Hospital of Bengbu Medical University, Bengbu, China; 2 Department of Radiology, Shanghai Municipal Hospital of Traditional Chinese Medicine, Affiliated with Shanghai University of Traditional Chinese Medicine, Shanghai, China; 3 Department of Radiology, The First Affiliated Hospital of Bengbu Medical University, Bengbu, China; 4 Department of General Surgery, The Second Affiliated Hospital of Bengbu Medical University, Bengbu, China

**Keywords:** breast cancer, pathological complete response, DCE-MRI, deep learning, 3D UNet

## Abstract

**Objective:**

The aim of this study is to develop a deep learning-based radiomic (DLR) model by fusing 3D features of tumor, peritumoral vessels, and metastatic lymph nodes from dynamic contrast-enhanced magnetic resonance imaging (DCE-MRI), with the goal of predicting pathological complete response (pCR) in breast cancer patients receiving neoadjuvant therapy.

**Materials and methods:**

A total of 200 breast cancer (BC) cases were retrospectively collected from the First and Second Affiliated Hospitals of Bengbu Medical University between January 2020 and December 2024. The cases were randomly allocated to a training set and a test set at a 1:1 ratio. For dynamic contrast-enhanced MRI (DCE-MRI) sequence imaging, 3D UNet technology was utilized to facilitate layer-by-layer semi-automated segmentation of tumors, peritumoral vessels, and metastatic lymph nodes. Concurrently, we used deep learning methods to extract features and constructed a predictive model for pCR status in breast cancer patients after NAT. The Clinical Combined Deep Learning Radiomic (CCDLR) model was developed by integrating clinical characteristics into the DLR model. The performance of the CCDLR and DLR models was compared and validated in a test set.

**Results:**

The training set contained 45 cases in the pCR group and 55 cases in the non-pCR group, while the test set contained 47 cases in the pCR group and 53 cases in the non-pCR group. The efficacy of the CCDLR model in predicting the NAT pCR of breast cancer was superior to that of the DLR model. The AUC values of the CCDLR model and the DLR model in the training set were 0.950 and 0.820, with accuracies of 96.0% and 81.0%, precision of 95.1% and 79.6%, recall of 95.1% and 84.3%, and F1 scores of 95.1% and 81.9%.In the test set, the AUC values of the two models were 0.870 and 0.850, with accuracies of 92.0% and 83.0%, precision of 92.1% and 83.3%, recall of 92.1% and 73.1%, and F1 scores of 92.1% and 77.9%.

**Conclusion:**

Fusing three-dimensional imaging features of tumors, peritumoral vessels, and metastatic lymph nodes, the DLR model shows favorable predictive efficacy. Importantly, the CCDLR model, constructed by incorporating clinical characteristics, exhibits significantly superior performance, underscoring its promising potential for clinical application in predicting pathological complete response (pCR) to neoadjuvant therapy (NAT) in breast cancer.

## 1 Introduction

Breast cancer (BC) is the most common malignant tumor in women, with a morbidity and mortality rate that are unparalleled among women on a global scale (1). The role of NAT in breast cancer treatment has been the subject of much research, with several studies indicating its potential to reduce tumor stage, increase the chances of surgical resection, and improve the rate of breast conservation (2–4). Additionally, it has been shown to avoid the unnecessary trauma caused by axillary lymph node dissection (5). The ultimate goal of NAT in BC patients is pCR (6), and the early prediction of NAT responsiveness and pCR status can assess the effectiveness of treatment and help clinicians adjust treatment regimens in a timely manner, thereby reducing the adverse consequences of unresponsive treatment (7). However, it should be noted that not all BC patients achieve pCR after NAT (8, 9). Furthermore, some studies (10) have demonstrated that there are discrepancies in the response to NAT among different tumor subtypes. In addition, due to the heterogeneity of the tumors, the individual response to NAT also varies. At present, the diagnosis of pCR is based on the postoperative resection of gross specimens and histopathological assessment (11). However, histopathological examination cannot provide a dynamic monitoring system for tumor responsiveness to NAT in real time. Furthermore, the assessment of pCR can only be carried out after the completion of NAT, resulting in a lag. Finally, surgical pathological resection is not easily repeatable (12). Consequently, the development of highly sensitive and specific prediction models for the early diagnosis of pCR status has become a significant research direction in the current field of BC precision medicine.

The concept of radiomics was first proposed by Lambin (13), with the objective being the extraction of quantitative features in a high-throughput manner and achieving the conversion from medical images to quantitative data. Feng et al. (14) investigated a machine learning model based on DCE-MRI for predicting NAT efficacy in BC, and the predictive ability of the model was not satisfactory. The construction of models for predicting pCR after NAT in BC patients is constrained by two limitations. Firstly, the majority of existing models focus on the intrinsic characteristics of the tumor (15) or simple binary analysis of “tumor-lymph node” failing to integrate the 3D imaging features of the primary tumor, peritumoral vessels and metastatic lymph nodes. Secondly, traditional radiomics relies on single-level Region of Interest (ROI) feature extraction, resulting in the loss of critical 3D spatial information. Of particular importance is that the radiological quantification of the peritumoral microenvironment has long been a research challenge. While studies have confirmed the impact of the peritumoral region on treatment response, components such as inflammatory cells are difficult to directly segment on conventional MRI. In contrast, peritumoral blood vessels, as “functional channels” for tumor invasion and metastasis and “visualizable markers” of microenvironmental status, have their morphology and density directly associated with tumor malignancy and drug delivery efficiency (12, 16). Abnormal peritumoral blood vessels can be clearly identified on DCE-MRI enhancement sequences, providing feasibility for their quantitative analysis. With the rapid advancement of deep learning and radiomics theories, the combined application and analysis of deep learning with key clinical characteristic information have further enhanced predictive capabilities (17). The DLR approach has broken through the conventional diagnostic and therapeutic frameworks in medicine, enabling in-depth mining of latent features and demonstrating exceptional performance (18).

The development of radiomics models based on three-dimensional imaging features of fused tumors, peritumoral vessels, and metastatic lymph nodes for predicting pCR after NAT in BC patients has not yet been systematically advanced. This research gap significantly limits the improvement of modeling based on spatial features of tumors and the ability to accurately predict prognosis. In order to address the aforementioned deficiencies, this study developed a DLR model that fuses 3D features of the tumor, peritumoral vessels, and metastatic lymph nodes in DCE-MRI images. This approach is especially important in reflecting the spatial variations of tumors. Specifically, the proposed methodology involves the segmentation of the tumor, peritumoral vessels, and metastatic lymph nodes in layers and the systematic analysis of their three-dimensional spatial distribution characteristics. This approach aims to overcome the limitations of traditional models in the integration of multidimensional tumor features and the use of spatial information. The objective is to more accurately capture three-dimensional geometric information, heterogeneity, and dynamic changes of the BC. The expected outcome is an improvement in the accuracy of the NAT efficacy prediction, thus providing a more reliable tool for the clinical prediction of the efficacy. The following text is intended to provide a comprehensive overview of the subject matter. In this study, we postulated that enhancing the diagnostic efficacy could be attained by constructing a prediction model that integrated the 3D features of these three elements. To this end, we retrospectively collected BC patients who underwent NAT and extracted the pre-NAT DCE-MRI images to obtain the deep learning features. Our objective was to construct a DLR model integrating 3D image features of the tumor, peritumoral vessels, and metastatic lymph nodes to predict BC patients’ post-NAT pCR.

## 2 Materials and methods

### 2.1 Patient population

**Figure 1:**
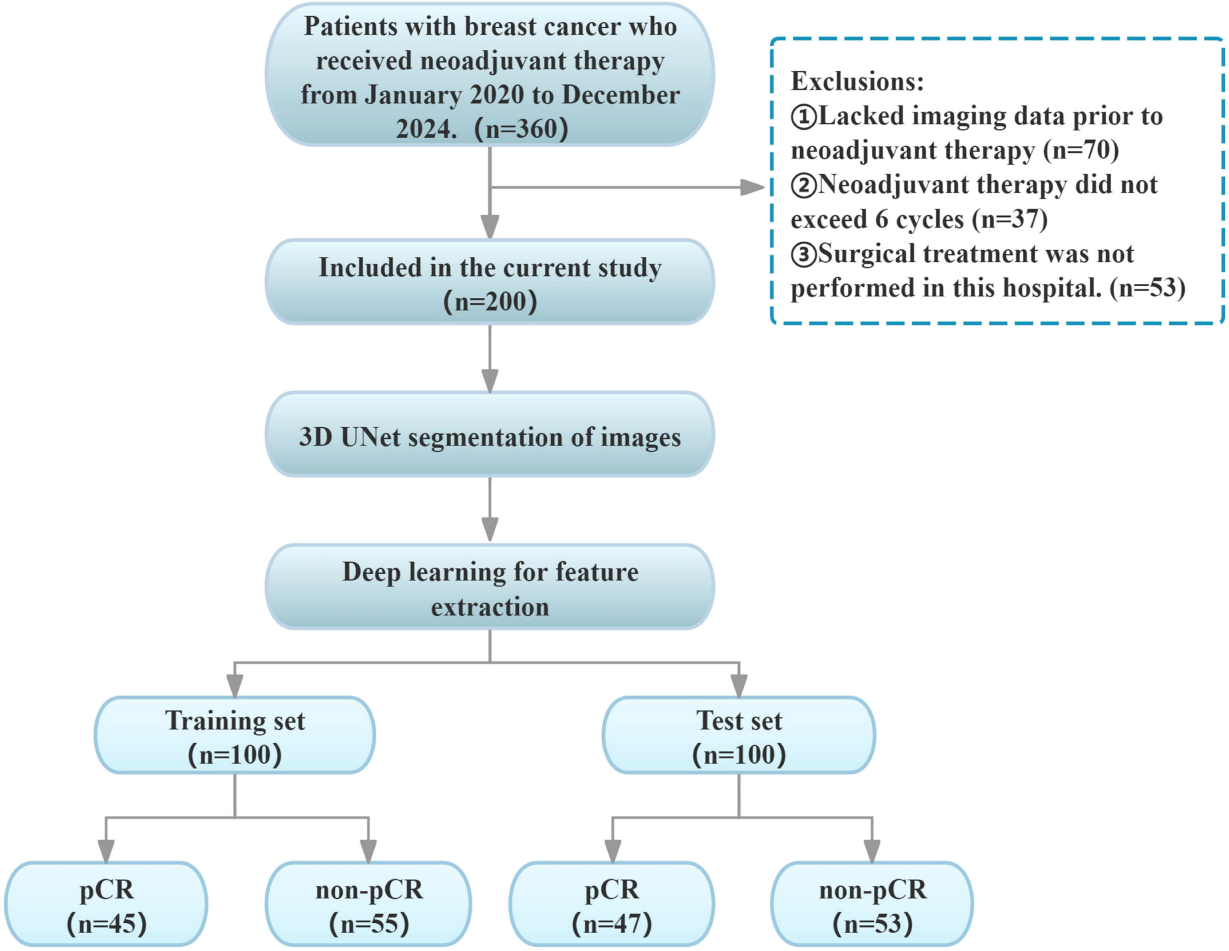
Enrolment pathway diagram.

A total of 200 breast cancer (BC) patients (mean age: 50.2 years, range: 26–79 years) were retrospectively enrolled by accessing the hospital information system (HIS) and picture archiving and communication system (PACS) between January 2020 and December 2024. Given the limited sample size, to balance the reliability of model training and validation while ensuring a sufficiently large independent test set for enhanced model robustness and credible generalization ability, this study adopted a two-stage validation design: 200 patients were randomly split into the training set (100 cases, 45 with pCR and 55 with non-pCR) and the independent test set (100 cases, 47 with pCR and 53 with non-pCR) at a 5:5 ratio. The test set was exclusively used for final evaluation, while five-fold cross-validation was applied to the training set to ensure training stability. The specific inclusion criteria were as follows: (1) Diagnosis of invasive breast cancer (BC) via puncture biopsy prior to neoadjuvant therapy (NAT);(2) No prior treatment received before NAT initiation;(3) Performance of breast dynamic contrast-enhanced MRI (DCE-MRI) before NAT;(4) Completion of a full NAT cycle (≥ 6 cycles); (5) Post-NAT surgical excision with pathological evaluation using the Miller-Payne (MP) pathological response grading system (19). Exclusion criteria were as follows: (1) Incomplete clinical or pathological data;(2) MRI images with severe artifacts or poor quality, compromising analysis;(3) History of concomitant malignant tumors. The present retrospective study was conducted in accordance with the principles of the Declaration of Helsinki, as approved by the Ethics Committee of the Affiliated Hospital of Bengbu Medical University. The study was exempted from the requirement of informed consent, as outlined in **Figure 1**.

### 2.2 Pathological classification and treatment protocols

The clinical data primarily encompassed patient demographics, menstrual status, tumor immunophenotyping, tumor location, maximum diameter (Measured based on pre-NAT contrast-enhanced DCE-MRI images), cystic necrosis, TIC type, adjacent chest wall invasion, skin invasion, axillary lymph node enlargement, clinical TN 23staging, and specific NAT regimen. Based on immunohistochemical (IHC) analysis, positivity criteria were defined as follows: ER/PR positivity: ≥1% of tumor cells with nuclear staining;HER2+: IHC 3+ or IHC 2+ confirmed by *in situ* hybridization (ISH) (20);Ki-67 high expression: proliferation index ≥20% (21).Tumor molecular subtypes were classified as per the following criteria (21): Luminal A: ER+,PR+, HER2-, and Ki-67<20%;Luminal B (HER2-): ER+, HER2-, and meeting at least one of the following criteria: Ki-67 ≥20%, low PR expression, or PR-;Luminal B (HER2+): ER+ and/or PR+, HER2+ (regardless of Ki-67 level); HER2 overexpression: ER-, PR-, HER2+; Triple-negative: ER-, PR-, HER2-. The chemotherapy regimens administered to the patients included in this study were all recommended in accordance with the guidelines of the National Comprehensive Cancer Network (NCCN) of the U.S. The chemotherapeutic agents employed primarily consisted of paclitaxel, anthracyclines, and platinum compounds. Hormone receptor-positive chemotherapy was administered in conjunction with endocrine therapy, while HER2+ patients received chemotherapy in conjunction with targeted (mono- or dual-antibody) therapy.

### 2.3 MRI acquisition

All DCE-MRI scans were completed within 1 week before the initiation of NAT. A Dutch Philips Achieva 3.0 T and an American GE Discovery 750W 3.0 T dual-gradient superconducting MRI scanner equipped with a SENSE 7-channel breast coil were used. Patients were positioned in the prone position with both breasts naturally and symmetrically falling into the coil, with the positioning center aligned with the center of the coil and the midpoint of the line connecting both nipples. Patients were instructed to minimize their respiratory rate to avoid motion artifacts caused by breathing and heartbeats. The DCE-MRI sequence parameters were as follows: TR, 4.5 ms; TE, 2.1 ms; FOV, 290 mm × 244 mm; matrix, 512 × 512; slice thickness, 1.2 mm; and slice spacing, 1.2 mm. Before dynamic contrast-enhanced scanning, a prescan image was acquired. Subsequently, gadolinium-based contrast agent (Gd-DTPA) was rapidly injected via an antecubital vein using a power injector at a dose of 0.2 mmol/kg and a flow rate of 2.0 mL/s, and scanning was initiated simultaneously. Eight dynamic image acquisitions were continuously performed after the injection of the contrast agent (each acquisition lasting 60 s, with a total duration of approximately 8 minutes). This design was intended to meet the needs of deep learning feature mining: compared with the conventional five-phase scanning in clinical practice, the eight time points with higher temporal resolution captured the complete perfusion process of the contrast agent “inflow–plateau–outflow,” providing a data basis for extracting fine hemodynamic features and assisting the model in identifying the dynamic differences related to treatment response in tumors, peritumoral vessels, and lymph nodes.

### 2.4 MRI image analysis and region of interest segmentation

Image analysis was conducted by two attending physicians with over a decade of experience in breast radiological diagnosis, utilizing the image PACS system. Disagreements in interpretation results were resolved through deliberation. The primary evaluation indices encompassed the following: tumor quadrant, maximum diameter, cystic necrosis, time-signal intensity curve (TIC) type, presence of axillary lymph node enlargement, chest wall invasion, skin invasion, and nipple indentation.

The target DCE-MRI raw images were retrieved from the PACS system, exported in DICOM standard format, and stored on the local hard drive. Subsequently, the image data was imported into the team’s in-house semi-automatic segmentation platform developed based on Python 3.8. This platform integrates the 3D U-Net architecture based on the mature open-source framework initially proposed by Çiçek et al. (Nature Methods, 2016) and utilizes the PyRadiomics 3.1.0 library for feature extraction. After importing the data, the region of interest (ROI) segmentation operation was performed in the software interface. First, all images underwent preprocessing as follows: (1) N4 bias field correction was performed using ANTs software to eliminate the impact of magnetic field inhomogeneity on model performance; (2) Resampling was conducted to a voxel size of 1 mm×1 mm×1 mm to mitigate spatial resolution discrepancies across different MRI scanners; (3) Z-score normalization was applied to eliminate gray intensity variations among various MRI devices. Segmentation of tumors, peritumoral blood vessels, and metastatic lymph nodes was completed by three radiologists proficient in breast disease diagnosis and skilled in the use of the segmentation software (1 with 3 years of experience and 2 with 10 years of experience). Blinded to patients’ clinical data and each other’s annotation results, the radiologists generated a three-dimensional volume of interest (VOI) by superimposing two-dimensional ROIs on each image slice. All three radiologists adhered to unified delineation criteria, avoiding obvious necrotic, hemorrhagic, and cystic areas during segmentation. To assess intra- and inter-observer reliability, manual independent segmentation of VOIs was performed for 30 randomly selected patients. Intra-class correlation coefficients (ICCs) were used to evaluate the consistency of results within the same observer and among different observers, with ICCs > 0.75 indicating good consistency. In cases of disagreement, consensus was reached through joint discussion or determined by a senior attending physician.

**Figure 2:**
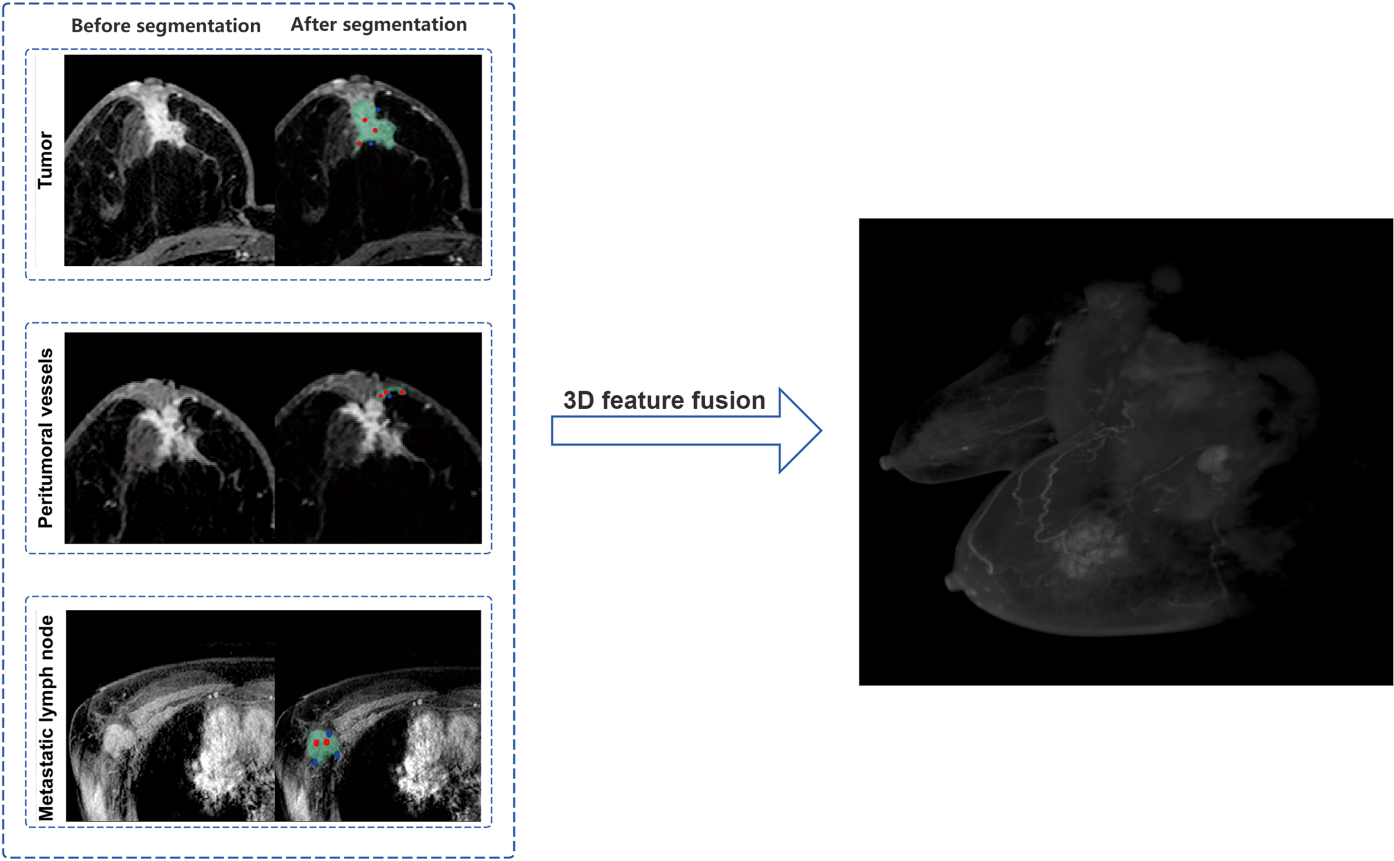
Schematic illustration of tumor, peritumoral vessels, and metastatic lymph nodes segmentation with 3D feature fusion.

Tumor Segmentation: The phase with the most significant enhancement was selected. Semi-automatic segmentation was performed, followed by manual boundary fine-tuning to completely delineate peripheral spiculations. peritumoral vessels Segmentation: Vessels with abnormal thickening (diameter ≥3 mm (16)) around the tumor were identified by comparison with the contralateral side and segmented slice by slice. Metastatic Lymph Node Segmentation: Lymph nodes with a short-axis diameter ≥1.5 cm or significant enhancement were selected for segmentation. In cases of multiple positive nodes, the largest one was chosen as the segmentation target. **Figure 2** shows the detailed segmentation of tumors, peritumoral vessels, and metastatic lymph nodes, as well as the schematic diagram of 3D feature fusion.

### 2.5 Feature extraction and selection

An interactive segmentation system was developed using the Dash framework, integrating deep learning-based interactive models (RITM and SAM) to construct a medical image segmentation visualization platform. By uploading multi-slice magnetic resonance (MR) images, the system supports user interaction via click-based point annotation or rectangular box drawing, enabling real-time updates of segmentation results. The front-end interface employs Plotly software for image visualization and interactive operations, including adding foreground/background points by clicking, rectangular annotation, and real-time parameter adjustment. The back-end architecture adopts multi-processing and Diskcache for callback task management, specifically designed to support complex model inference.

The system offers two segmentation interaction modes: the RITM model generates interaction points through user clicks to iteratively optimize segmentation results, while the SAM model performs one-time inference via click points or bounding boxes to produce high-quality masks. The generated masks are grouped, labeled, and visualized, and can be downloaded as image files. Three-dimensional (3D) models are reconstructed based on the segmentation masks of each slice, and 3D radiomic features are extracted using the PyRadiomics 3.1.0 toolkit.

**Figure 3:**
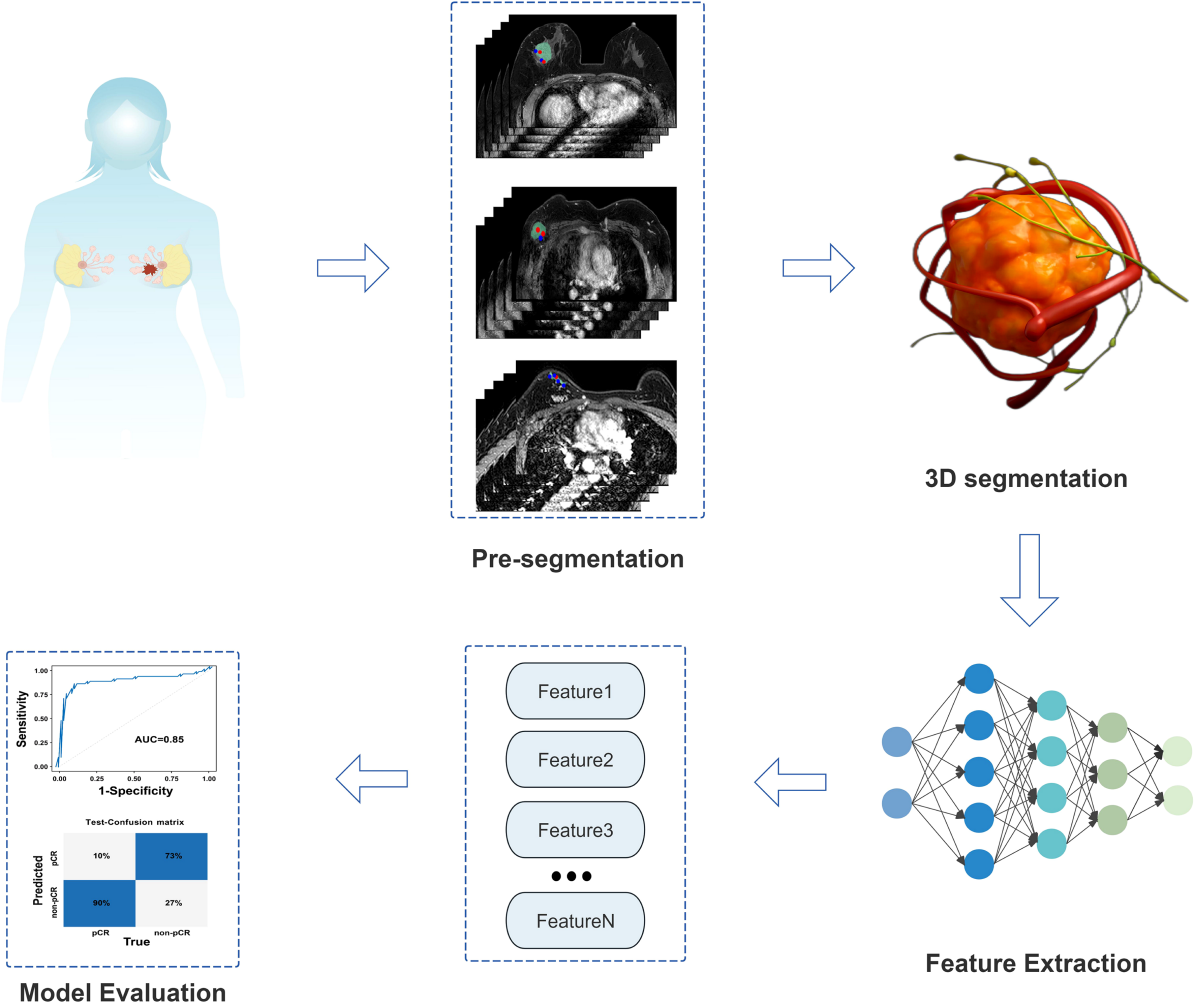
Technical roadmap flowchart of the DLR model.

Specifically, to accurately segment the 3D volumes of tumors, peritumoral blood vessels, and metastatic lymph nodes, we adopted a standard 4-layer encoder-decoder 3D UNet model. The encoder path of the network extracts hierarchical features through four downsampling modules, each consisting of two consecutive 3x3x3 convolutional layers and one 2x2x2 max-pooling layer. The number of feature channels is doubled incrementally from the initial 32 to 256. In the decoder path, upsampling is performed via transposed convolution, and skip connections are used to concatenate feature maps from corresponding levels of the encoder with upsampled feature maps, restoring high-resolution detailed information. ReLU is employed as the activation function after all convolutional layers within the network, while the final output layer uses the Sigmoid activation function to generate a probability map indicating the likelihood of each voxel belonging to the target region. During the training phase, the AdamW optimizer was selected with an initial learning rate of 1e-4, combined with a cosine annealing learning rate scheduling strategy for dynamic adjustment. To address the common foreground-background imbalance in medical images and stabilize the training process, the loss function adopts a weighted combination of Dice loss and binary cross-entropy loss (50% each). The entire training process is set to a maximum of 200 epochs, with an early stopping mechanism based on the Dice coefficient of the test set: training is automatically terminated if performance does not improve for 20 consecutive epochs to prevent overfitting and ensure the selection of the optimal-performing model. Based on the 3D regions of tumors, peritumoral blood vessels, and metastatic lymph nodes segmented by the aforementioned 3D UNet model, we further extracted relevant radiomic features and performed rigorous feature selection via LASSO regression, ultimately obtaining 65 most informative features. These features integrate morphological, intensity, and texture information of tumors, peritumoral blood vessels, and metastatic lymph nodes, and are used as the core input for subsequent construction of the deep learning regression (DLR) model **Figure 3**.

### 2.6 Statistical analysis

The statistical analysis of the data was conducted utilizing the SPSS 27.0, Medcalc 20.0.22 and R studio software, employing the χ² test or Fisher’s exact test for categorical variables between the two groups. The Shapiro-Wilk normality test was employed for continuous variables, and continuous variables conforming to the normal distribution were denoted by $\bar{x}\pm s$, with an independent samples *t* test. In order to ascertain the independent influences, it was necessary to submit the statistically different variables (*P<* 0.05) to binary logistic regression analysis following one-way analysis of variance. Receiver operating characteristic (ROC) curves were plotted to quantify the classification performance of the models, and the DeLong test was used for statistical comparison of differences in the area under the curve (AUC) between different models or methods. Second, AUC, accuracy, precision, recall, and F1-score were calculated. All metrics were subjected to 10,000 Bootstrap resamplings to compute 95% confidence intervals (CIs) for reflecting estimation accuracy. Calibration curves were plotted to intuitively demonstrate the consistency between the predicted probabilities of the models and the actual occurrence probabilities. Finally, decision curve analysis (DCA) was applied to evaluate the clinical net benefit of the prediction models at different decision thresholds, intuitively reflecting their potential utility in aiding clinical decision-making. A two-tailed P-value< 0.05 was considered statistically significant.

## 3 Results

### 3.1 Clinical characteristics

**Figure 4:**
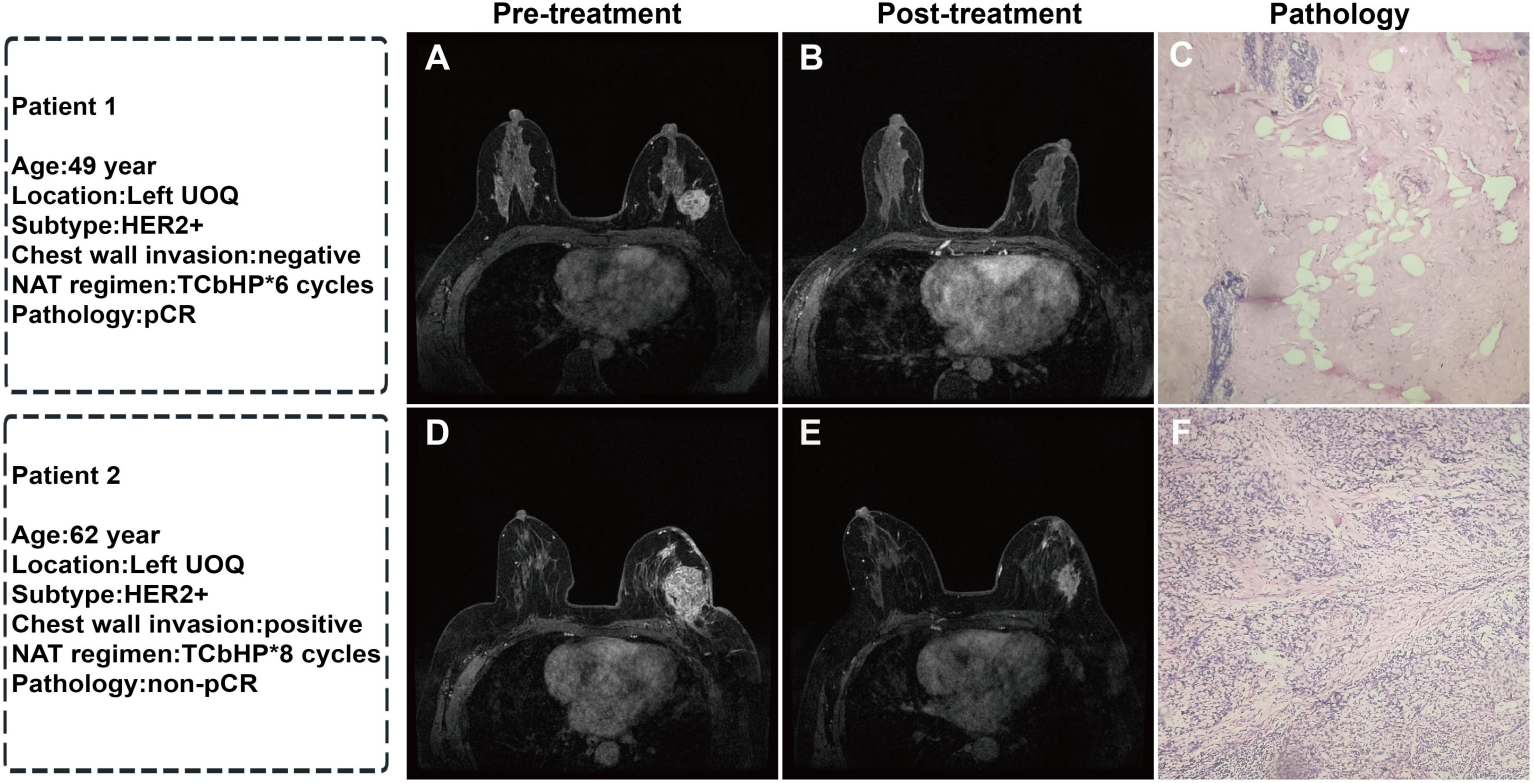
Representative MRI images before and after NAT and corresponding postoperative pathological sections in two breast cancer patients: **(A)** Pre-treatment MRI enhancement image of the left breast tumor; **(B)** Post-NAT MRI image showing complete disappearance of the tumor; **(C)** Postoperative pathological section (HE staining, ×40) showing no residual cancer cells, Miller-Payne grade 5 (pCR); **(D)** Pre-treatment MRI enhancement image of the left breast tumor; **(E)** Post-NAT MRI image showing a reduction in tumor size; **(F)** Postoperative pathological section (HE staining, ×40) showing residual cancer cells, Miller-Payne grade 3 (non-pCR). (UOQ: upper outer quadrant; TCbHP: docetaxel + carboplatin + trastuzumab + pertuzumab).

Among 200 breast cancer (BC) patients, 92 cases were classified into the pCR group and 108 into the non-pCR group. Univariate analysis identified statistically significant associations (*P<* 0.05) with PR status, HER2 expression, molecular subtype, N stage, chest wall invasion, and administration of paclitaxel, anthracycline, platinum-based chemotherapy, as well as endocrine/targeted therapies (**Table 1**). Multivariate logistic analysis revealed that HER2 expression and chest wall invasion were independent predictors of pCR following neoadjuvant therapy (NAT) in BC (*P<* 0.05). Specifically, BC patients with HER2+ and absence of chest wall invasion demonstrated a significantly higher likelihood of achieving pCR after NAT (**Table 1**, **Figure 4**).

**Table 1:** Univariate and multivariate analyses of clinical characteristics in 200 enrolled BC patients.

Characteristics	Single factor			Multifactor	
	non-pCR (n=108)	pCR (n=92)	P value	OR (95% CI)	P value
Age/years ^a^	49.39 ± 9.84	51.07 ± 9.65	0.071		
Menstruation ^b^			0.860		
Premenopausal	53 (49.1)	44(47.8)			
Postmenopausal	55 (50.9)	48(52.2)			
ER status ^b^			0.077		
Negative	44 (40.7)	49(53.3)			
Positive	64 (59.3)	43(46.7)			
PR status ^b^			**0.002**	0.673(0.272~1.660)	0.390
Negative	60 (55.6)	70(76.1)			
Positive	48 (44.4)	22(23.9)			
HER2 status ^b^			**<0.001**	2.638(1.025~ 6.785)	**0.044**
Negative	61 (56.5)	24(26.1)			
Positive	47 (43.5)	68(73.9)			
Ki-67 level ^b^			0.056		
<20%	11 (10.2)	3(3.3)			
≥20%	97 (89.8)	89(96.7)			
Molecular subtypes ^c^			**0.023**	1.089(0.627~1.889)	0.763
Luminal A	7 (6.5)	0(0.0)			
Luminal B	58 (53.7)	44(47.8)			
HER2 overexpression	27 (25.0)	36(39.1)			
Triple-negative	16 (14.8)	12(13.0)			
Clinical T stage ^b^			0.622		
T1	7 (6.5)	9(9.8)			
T2	70 (64.8)	63(68.5)			
T3	24 (22.2)	16(17.4)			
T4	7 (6.5)	4(4.3)			
Clinical N stage ^b^			**0.019**	0.769 (0.516-1.146)	0.196
N0	9 (8.3)	7(7.6)			
N1	40 (37.0)	43(46.7)			
N2	41 (38.0)	39(42.4)			
N3	18 (16.7)	3(3.3)			
NAT drugs ^b^					
Paclitaxel			**0.038**	0.261(0.061~1.119)	0.071
No	3 (2.8)	9(9.8)			
Yes	105 (97.2)	83(90.2)			
Anthracycline			**<0.001**	1.404(0.237~8.303)	0.708
No	52 (48.1)	67(56.3)			
Yes	56 (51.9)	25(30.9)			
Platinum			**0.026**	0.977(0.367~2.603)	0.963
No	64 (61.5)	40(38.5)			
Yes	44 (45.8)	52(54.2)			
Endocrine or targeted therapy			**<0.001**	2.026(0.380~10.812)	0.408
No	59 (70.2)	25(29.8)			
Yes	49 (42.2)	67(57.8)			
Maximum tumor diameter ^a^	34.15± 15.60	31.99± 14.96	0.207		
Tumor quadrant ^b^			0.123		
UOQ	58 (53.7)	53(57.6)			
LOQ	15 (13.9)	4(4.3)			
LIQ	7 (6.5)	7(7.6)			
UIQ	15 (13.9)	20(21.7)			
Central District	13 (12.0)	8(8.7)			
Cystic necrosis ^b^			0.052		
No	62 (57.4)	65(70.7)			
Yes	46 (42.6)	27(29.3)			
TIC type ^b^			0.228		
Inflow type	43 (39.8)	26(28.3)			
Outflow type	46 (42.6)	46(50.0)			
Platform type	19 (17.6)	20(21.7)			
Axillary lymphadenopathy ^b^			0.704		
No	20 (18.5)	19(20.7)			
Yes	88 (81.5)	73(79.3)			
Chest wall invasion ^b^			**0.006**	0.374(0.165~0.847)	**0.018**
No	78 (72.2)	81(88.0)			
Yes	30 (27.8)	11(12.0)			
Skin invasion ^b^			0.324		
No	43 (39.8)	43(46.7)			
Yes	65 (60.2)	49(53.3)			
Nipple retraction ^b^			0.133		
No	73 (67.6)	71(77.2)			
Yes	35 (32.4)	21(22.8)			

Continuous variables are presented as mean ± standard deviation ( $\bar{x}\pm s$) and categorical variables as number (%) of cases. BC, breast cancer; ER, estrogen receptor; PR, progesterone receptor; HER2, human epidermal growth factor receptor 2; UOQ, upper outer quadrant; LOQ, lower outer quadrant; LIQ, lower inner quadrant; UIQ, upper inner quadrant; TIC, time-intensity curve. Statistical analyses included: a) independent samples *t*-test for continuous variables with normal distribution; b) chi-square test for categorical variables with expected frequencies ≥5; c) Fisher’s exact test for categorical variables with expected frequencies<5. Bold text indicates statistical significance (*P<* 0.05).

### 3.2 Construction of the DLR model

To mitigate the risk of overfitting, L2 regularization (weight decay coefficient of 1e-5) was incorporated into the model to constrain the parameter scale. Five-fold cross-validation was applied to the training set, ensuring training stability through multiple rounds of iteration.

**Figure 5:**
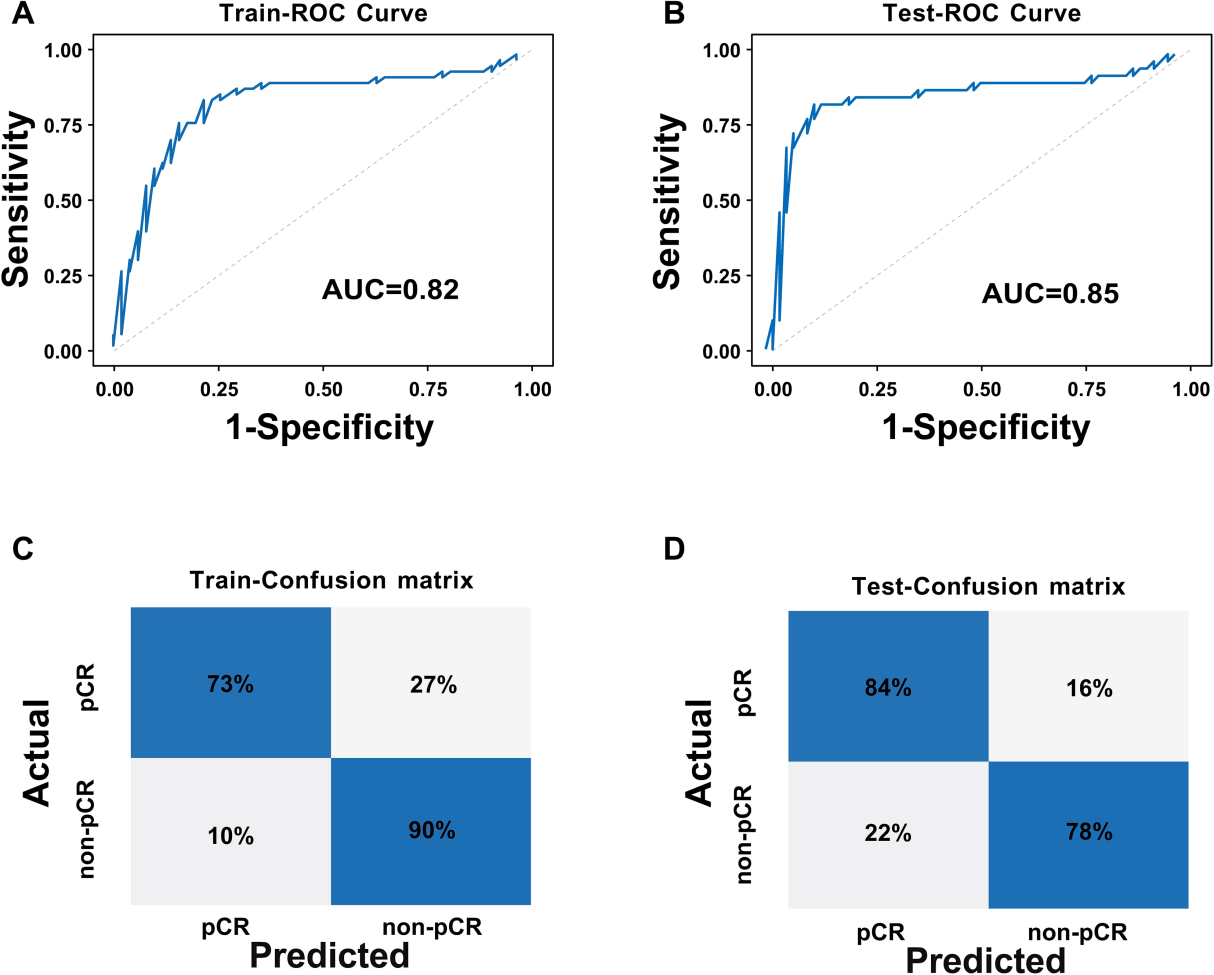
ROC curves and confusion matrices of the DLR model: **(A)** ROC curve of the training set; **(B)** ROC curve of the test set; **(C)** Confusion matrix of the training set; **(D)** Confusion matrix of the test set.

The DLR model was constructed based on 65 core three-dimensional deep learning features screened from DCE-MRI images, which comprehensively cover three key dimensions: morphological features (e.g. original_shape_Sphericity, original_shape_VoxelVolume) that quantify the spatial structural characteristics of tumors, peritumoral vessels, and metastatic lymph nodes; first-order intensity features (e.g. original_firstorder_Mean, original_firstorder_Variance) that reflect the dynamic distribution pattern of contrast agent perfusion; and texture features (e.g. original_glrlm_RunEntropy, original_glszm_ZoneVariance) that characterize the heterogeneity of target regions. Collectively, these features establish a quantitative description system for the “tumor-peritumoral microenvironment-metastasis” axis, enabling accurate capture of subtle biological changes invisible to the naked eye during treatment response. The area under the curve (AUC) values of the DLR model in the training set and test set were 0.82 and 0.85. Respectively, the DLR model demonstrated strong performance in both sets, with high accuracy, precision, recall, and F1 scores (**Table 2**, **Figure 5**). This indicates that the model performs well in terms of sample identification and differentiation, and is able to better discriminate between positive and negative samples in terms of diagnostic accuracy and stability. This has potential clinical application value and reliability.

**Table 2:** Efficacy comparison between the DLR and CCDLR models.

	AUC (95%*CI*)	Accuracy	Precision	Recall	F1 Score
Train					
DLR model	0.820(0.750~0.900)	0.810	0.796	0.843	0.819
CCDLR model	0.950(0.920~0.980)	0.960	0.951	0.951	0.951
Test					
DLR model	0.850(0.730~0.950)	0.830	0.833	0.731	0.779
CCDLR model	0.870(0.790~0.930)	0.920	0.921	0.921	0.921

AUC, area under the curve; *CI*, confidence interval

### 3.3 Construction of the CCDLR model

**Figure 6:**
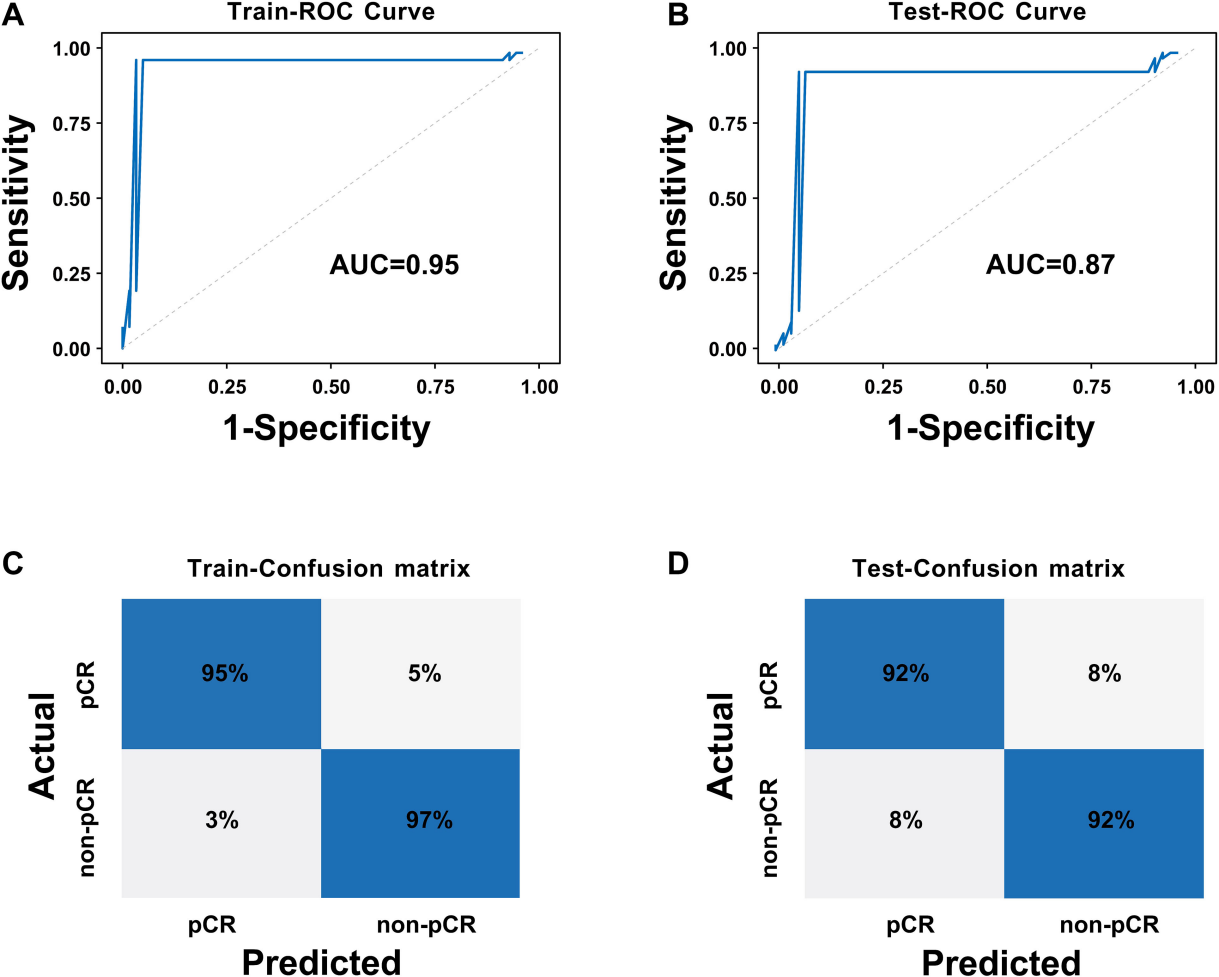
ROC curves and confusion matrices of the CCDLR model: **(A)** ROC curve of the training set; **(B)** ROC curve of the test set; **(C)** Confusion matrix of the training set; **(D)** Confusion matrix of the test set.

To achieve effective fusion of clinical features and deep learning features while avoiding model overfitting and variable redundancy, we first performed univariate and multivariate Logistic regression analyses based on clinical data (**Table 1**) to systematically screen for independent predictors of pCR. The results revealed that HER2 expression status and chest wall invasion were the only independent clinical predictors validated by statistics. Based on the aforementioned screening results, we constructed the CCDLR model by further integrating these two independent clinical factors (HER2 status and chest wall invasion) into the 65 core deep learning features of the DLR model. The performance of the CCDLR model was superior to that of the DLR model. The CCDLR model exhibited higher AUC values, accuracy, precision, recall, and F1 scores than the DLR model in both the training and test sets (**Table 2**, **Figure 6**).

**Figure 7:**
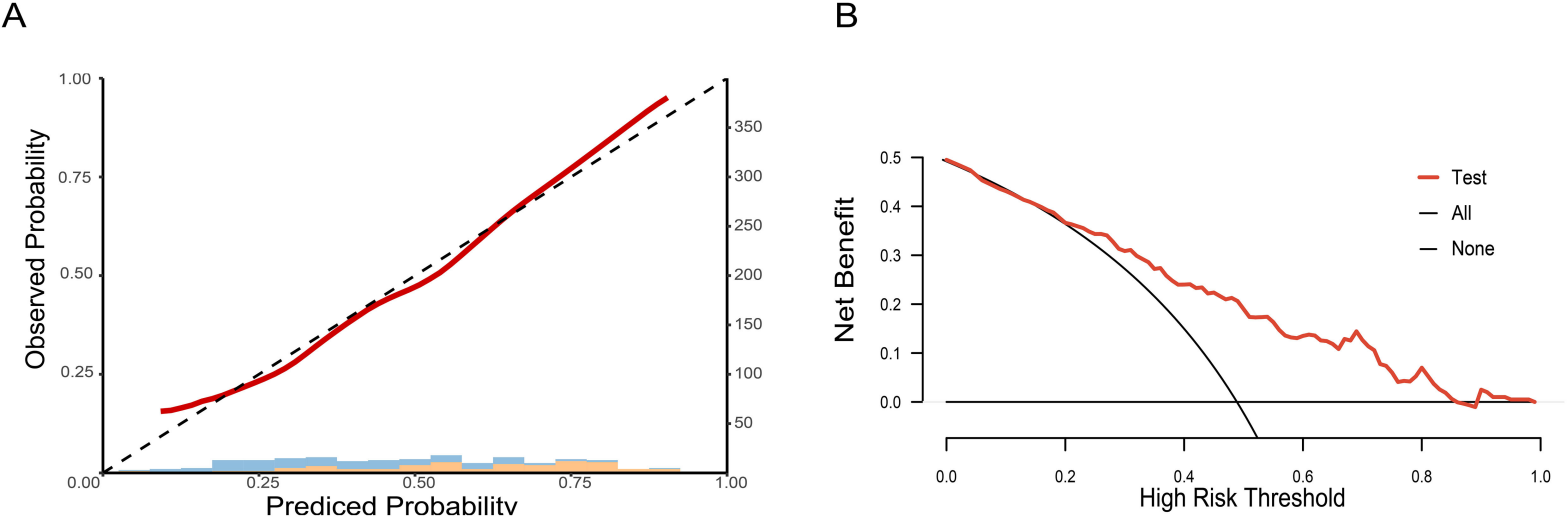
Calibration curve and decision curve of the CCDLR model in the test set: **(A)** Calibration curve; **(B)** Decision curve.

To clarify the predictive reliability and clinical utility of the CCDLR model in independent samples, this study evaluated the consistency between the model’s predicted probabilities and the actual pathological complete response (pCR) rate in the test set using calibration curves. The clinical net benefit was quantified via Decision Curve Analysis (DCA), with results shown in **Figure 7**.

The DeLong test revealed that the differences in performance between the CCDLR model and the DLR model were statistically significant in both the training and test sets (P< 0.05), with the CCDLR model demonstrating superior diagnostic performance. The calibration curve (**Figure 7A**) indicated good consistency between the CCDLR model’s predictions of pCR status across all cohorts and the ideal model (P > 0.05). DCA (**Figure 7B**) suggested that the CCDLR model had high clinical applicability.

## 4 Discussion

This study aims to develop a non-invasive preoperative prediction model for evaluating NAT sensitivity in breast cancer (BC) patients, with the goal of assisting clinicians in formulating personalized treatment plans to maximize therapeutic efficacy. Preoperative neoadjuvant therapy (NAT) plays a central role in the standardized management of breast cancer, where escalation and de-escalation strategies serve as its core components. Escalation strategies are primarily tailored for high-risk patients or those with suboptimal treatment responses, where treatment intensity is escalated to enhance therapeutic outcomes. In clinical practice, HER2+ patients who demonstrate a poor response to conventional NAT should be administered dual-targeted agents or platinum-based chemotherapy. Conversely, de-escalation strategies are predominantly used for low-risk patients or those with favorable responses, aiming to minimize unnecessary chemotherapy-related side effects and improve care quality by de-escalating treatment intensity. This approach thus has the potential to enhance patients’ quality of life by reducing chemotherapy-associated adverse effects. For early-stage HER2+ breast cancer patients with low tumor burden and no lymph node metastasis, consideration may be given to implementing a regimen consisting of dual-targeted therapy alone, without the inclusion of chemotherapeutic agents (22). It is imperative to accurately assess NAT response status in breast cancer (BC) patients and implement escalation or de-escalation strategies. This approach ensures treatment efficacy while minimizing chemotherapy-related toxicities, thereby enhancing the precision and personalization of therapy (23).

In this study, a preoperative prediction model for assessing pathological complete response (pCR) status following neoadjuvant therapy (NAT) in breast cancer (BC) patients was developed. The model was based on a Deep Learning Radiomic (DLR) framework constructed by fusing 3D features from DCE-MRI of tumors, peritumoral vessels, and metastatic lymph nodes. First, for segmentation of the region of interest (ROI) in DCE-MRI images, the traditional manual segmentation method was eschewed in favor of importing DCE-MRI data into 3D UNet semi-automatic segmentation software, aiming to initially segment images rapidly. The 3D UNet semi-automatic process was a rapid, automated procedure that facilitates ROI delineation by establishing a gray-scale threshold and adjusting the gray-value range”. Subsequently, manual refinement of the delineation was performed to ensure boundary accuracy and precise representation of burrs and surface irregularities. Following this, the segmented mask was uploaded to PyRadiomics 3.1.0 software to extract features in a unified format. The 3D UNet semi-automatic segmentation reduces dependency on clinician experience, mitigates errors from subjective factors, and significantly enhances segmentation efficiency and accuracy. The development of the CCDLR model involved integrating two components: the DLR model (based on DCE-MRI feature extraction) and clinical features. This integration was undertaken to enhance model performance. Analysis results showed that the CCDLR model exhibited significantly improved AUC, accuracy, precision, recall, and F1-score compared to the DLR mode in both training and test sets. The findings demonstrate that the CCDLR model possesses enhanced stability, superior diagnostic accuracy, effective sample discrimination, reliable positive/negative sample identification, and clinical applicability for predicting pCR status after NAT in BC patients.

This study proposes a deep learning model for predicting post-neoadjuvant therapy (NAT) pathological complete response (pCR) in breast cancer (BC) patients, offering two significant advantages over other radiomics models based on MRI feature extraction. First, our research extends beyond tumor-centric characteristics by exploring the potential of broadening ROI extraction to include peritumoral vessels and distant metastatic lymph nodes. Multi-dimensional tumor features are integrated and analyzed in three dimensions to capture the spatial architecture, deep heterogeneity, and dynamic changes of BC, enabling comprehensive analysis of tumor-microenvironment interactions. Although traditional studies have recognized the impact of the peritumoral space on treatment response, most have struggled to incorporate it into models due to the lack of clear imaging quantification standards for components such as inflammatory cells (24). In this study, we specifically selected peritumoral blood vessels as a “surrogate marker” for the peritumoral microenvironment, which is based on both biological evidence and clinical feasibility. Biologically, peritumoral blood vessels serve as critical carriers for tumors to obtain nutrients and facilitate invasion and metastasis, and their abnormal morphology can reflect treatment sensitivity (12). Clinically, peritumoral blood vessels can be preliminarily segmented on DCE-MRI, avoiding the technical barriers associated with the quantification of inflammatory cells. By integrating the 3D features of peritumoral blood vessels, tumors, and lymph nodes, the model can more comprehensively analyze the interactions within the “tumor-microenvironment-metastasis” axis and identify subtle abnormalities that are overlooked in single tumor analysis—this is also one of the core reasons for its superior performance compared to traditional models. In a study by Sunghoon et al. (3), 3D MR images of bilateral axillae and chest wall were integrated, showing that whole-image-trained models outperformed cropped MRI counterparts. Second, this study achieves volume of interest (VOI) segmentation and feature extraction via layer-by-layer outlining, overcoming limitations of traditional 2D single-slice analysis to fully exploit 3D stereoscopic features. Feng et al. (14) investigated an integrated model combining ultrasound multi-phase 2D image features with clinical data for post-NAT pCR prediction in BC, noting moderate performance discrepancy between training and test sets. The use of 3D radiomics features was identified as a key factor in enhancing model efficacy, as they enable comprehensive tumor representation—unlike 2D features—to capture heterogeneity. Netti et al. (24) showed that models based on 3D radiomics features exhibited enhanced stability without increasing residual correlation heterogeneity. Thus, this study employs 3D feature extraction for model construction: the DLR model integrates tumor, peritumoral vessel, and metastatic lymph node 3D imaging characteristics, combining advanced imaging technology with deep learning algorithms for in-depth, comprehensive analysis.

In the clinical multivariate analysis, this study identified HER2 expression and chest wall invasion as independent predictors of pCR following neoadjuvant therapy (NAT) in breast cancer. HER2+ BC patients demonstrated a significantly higher likelihood of achieving pCR after NAT, consistent with Pu et al. (25), who reported an odds ratio (OR) of 1.833 for HER2+ in attaining pCR. Notably, the pCR group in our cohort exhibited a substantially higher proportion of HER2+ cases (73.9% vs. 43.5%, *P* < 0.001). HER2 represents a pivotal therapeutic target for precision medicine (19), is central to this association: clinical applications of HER2-directed agents (trastuzumab and pertuzumab) have been shown to significantly enhance pCR rates in HER2+ BC, thereby improving patient prognosis and quality of life. Logistic regression analysis further revealed that BC patients without chest wall invasion were more likely to achieve pCR after NAT. In our cohort of 200 patients, the pCR group had a higher proportion of chest wall invasion-negative cases (88.0% vs. 72.2%, *P* = 0.006), aligning with prior findings (26) that chest wall invasion is a hallmark of locally advanced BC associated with suboptimal treatment response. This may be attributed to the aggressive biology of chest wall-invasive tumors, which exhibit rapid progression and reduced sensitivity to NAT (27). Notably, a recent study (28) demonstrated that radical surgery combined with chest wall radiotherapy can improve cure rates and survival outcomes in such patients, underscoring the importance of integrating anatomical invasion status into treatment stratification.

This study has the following limitations: First, potential errors may exist in image segmentation. Although the semi-automated segmentation using 3D UNet was annotated slice-by-slice by three specialist radiologists and reviewed and calibrated by chief physicians, it still faces challenges related to subjective judgment in practical operations. Regarding the definition of peritumoral blood vessels, although the standard of “diameter ≥ 3 mm” was adopted with reference to the contralateral normal blood vessels, issues such as abnormal vascular morphology and blurred enhancement boundaries caused by tumor infiltration may lead to deviations in the inclusion or exclusion of borderline blood vessels. In terms of distinguishing metastatic lymph nodes, it is difficult to completely differentiate metastatic lymph nodes from reactive hyperplastic lymph nodes solely based on the imaging features of “short-axis diameter ≥ 1.5 cm or obvious enhancement”. The aforementioned segmentation deviations may result in inaccurate extraction of 3D features of tumors, peritumoral blood vessels, and metastatic lymph nodes, thereby affecting the reliability of the input data for the model.

Second, there is a limitation in data centers. The geographical homogeneity of patients from the two centers may restrict the cross-regional generalization of the model. Targeted optimizations will be conducted in the future: developing a multimodal combined segmentation model of DCE-MRI + DWI, establishing a multi-center cohort of more than 400 cases by collaborating with multiple hospitals, and collecting MRI data before, during, and after NAT to develop a dynamic prediction model. Third, the pCR rate observed in this cohort (46.0%, 92/200) is relatively high. Specifically, the proportion of HER2-positive patients in the pCR group reached 73.9% (68/92), which was significantly higher than the 43.5% (47/108) in the non-pCR group. It is known that HER2 positivity is a well-established predictive factor for a favorable response to neoadjuvant therapy (19, 25), and the high proportion of patients with this subtype may have elevated the overall pCR rate. In addition, most HER2-positive patients received a dual-targeted therapy (trastuzumab combined with pertuzumab) plus chemotherapy regimen. Although this potent treatment strategy is in line with clinical guidelines, it has further increased the incidence of pCR (22, 23). Such biases in patient subtypes and treatment regimens may limit the universality of the model, especially in clinical scenarios where the proportion of HER2-positive patients is low or the treatment regimens are relatively simplified. Future studies should include cohorts with more balanced molecular subtypes and treatment regimens to verify the broad applicability of the model. Fourth, the classification of ER expression did not further subdivide the low-expression subtype. Although this study defined ER positivity as ER ≥ 1% in accordance with standard criteria, cases with low ER expression (1%-10%) exhibit unique biological behaviors and treatment response characteristics (29). In this study, there were relatively few cases of low ER expression. The insufficient sample size prevented separate analysis of its impact on pCR prediction and failed to assess whether such patients require differentiated treatment decisions. Future studies need to further expand the sample size and subdivide ER expression into three grades: “negative (<1%)”, “low expression (1%-10%)”, and “high expression (>10%)” to further verify the independent predictive value of the low-expression subtype and provide a basis for clinical stratified treatment.

In summary, we employed 3D UNet semi-automatic segmentation technology and a deep learning method, fused 3D image features of the tumor, peritumoral vessels, and metastatic lymph nodes, and combined these with clinical features to successfully develop a CCDLR model to predict post-NAT pCR in BC patients. The CCDLR model demonstrates superior performance, facilitates clinical decision-making, and possesses the potential for clinical application in predicting NAT pCR in BC patients.

## Data Availability

The original contributions presented in the study are included in the article/supplementary material. Further inquiries can be directed to the corresponding authors.
